# Clinical characteristics and treatment outcomes of tuberculous tenosynovitis of the hand and wrist

**DOI:** 10.7150/ijms.84727

**Published:** 2023-05-21

**Authors:** Cao Thanh Ngoc, Nguyen Chau Tuan, Nguyen Phuc Thinh, Nguyen Minh Duc

**Affiliations:** 1 Department of Geriatrics and Gerontology, University of Medicine and Pharmacy at Ho Chi Minh City, Vietnam.; 2 Department of Rheumatology, University Medical Center HCMC, Ho Chi Minh City, Vietnam.; 3 Department of Orthopedics, University Medical Center HCMC, Ho Chi Minh City, Vietnam.; 4 Department of Radiology, Pham Ngoc Thach University of Medicine, Ho Chi Minh City, Vietnam.

**Keywords:** tuberculosis, tenosynovitis, wrist, hand

## Abstract

**Objective**: Vietnam is endemic with tuberculosis (TB), which is highly prevalent in the community. TB tenosynovitis of the wrist and hand is uncommon. Because of its insidious progression and atypical presentations, it is often difficult to diagnose, leading to treatment delays. This study investigates the characteristics of clinical and subclinical signs and treatment outcomes of patients with TB tenosynovitis in Vietnam.

**Patients and Methods**: This prospective longitudinal cross-sectional study included 25 TB tenosynovitis patients in the Rheumatology Clinic at University Medical Center Ho Chi Minh City. The diagnosis was made based on a tuberculous cyst in histopathological specimens. The data were collected through medical history, physical examination, and medical records, including demographics, signs, symptoms, condition duration, and related laboratory tests and imaging. The outcomes of all participants were assessed after 12 months of treatment.

**Results**: The most common symptom of TB tenosynovitis was swelling of the hand and wrist, which was present in all patients. Its other symptoms included mild pain and numbness of the hand in 72% and 24% of patients, respectively. It can affect any site on the hand. Hand ultrasound findings included thickening of the synovial membrane (80%), peritendinous effusion (64%), and soft tissue swelling (88%). Most patients (18/22) had a good outcome after the treatment with anti-tubercular drugs.

**Conclusions**: TB tenosynovitis progression is often insidious. Its most common symptoms are swelling of the hand and mild pain. Ultrasound is a useful tool to support the diagnosis. A histological examination confirms the diagnosis. Most cases respond and have a good outcome after 9-12 months of anti-tuberculosis treatment.

## Introduction

Tuberculosis (TB) infections remain a significant cause of morbidity and mortality worldwide.^1^ According to the World Health Organization, TB is one of the ten leading causes of death and the leading cause of infectious diseases caused by a single agent.^2^ In 2021, an estimated 10.6 million people were infected with TB and 1.6 million died from TB worldwide. In addition, TB incidence increased by 3.6% in 2021 compared to 2020^3^. Despite preventive and curative measures, TB remains a burden on healthcare systems.

TB can affect any organ in the body, with variable manifestations in each system. Musculoskeletal system involvement accounts for approximately 10% of extrapulmonary TB manifestations.^4^ The presentation of musculoskeletal TB may be insidious over a long period and the diagnosis may be delayed, as TB may not be the initial consideration in differential diagnosis. Besides, concomitant pulmonary involvement may not be present, contributing to confuse the diagnosis even further.^5^

TB tenosynovitis of the wrist and hand is an unusual infection of the tendon sheath and synovial membrane of the wrist and hand. This rare manifestation accounts for only about 5% of TB cases.^6^ Because of its insidious course and atypical presentation, it can be misdiagnosed as other diseases, such as negative rheumatoid arthritis, De Quervain's tendonitis, and carpal tunnel syndrome.^7^,^8^ These factors contribute to difficulty in diagnosis and delays in treatment, leading to many severe consequences and complications such as erosive bone and disability.^7^

Vietnam is endemic with TB and has an overall estimated prevalence of bacteriologically-confirmed TB among adults of 322 per 100,000 population.^1^ With atypical manifestations, which can be confused with many other diseases, TB tenosynovitis poses significant challenges in diagnosis. In addition, waiting for histopathologic results confirming the diagnosis can delay treatment, resulting in many complications for patients.^9^ Therefore, we aimed to investigate the main characteristics of clinical and subclinical signs and treatment outcomes of patients with TB tenosynovitis. This study can contribute to a deeper understanding of TB tenosynovitis manifestations to assist in its early diagnosis and adequate treatment for all patients.

## Patients and Methods

### Study location

This prospective longitudinal cross-sectional study selected participants from among TB tenosynovitis patients visiting the Rheumatology Clinic at the University Medical Center Ho Chi Minh City between October 2020 and June 2021. The TB tenosynovitis diagnosis was made based on the presence of tuberculous cysts in histopathological specimens. An orthopedic surgeon conducted the synovial biopsy at the University Medical Center, and the histopathological result was analyzed at Pham Ngoc Thach Hospital. Patients with a history of hand trauma, previous hand surgery, peripheral neuropathy, and mental health were excluded. This study was approved by the Institution Review Board of the University Medical Center Ho Chi Minh City.

### Data collection

We collected participants' demographic characteristics from their electronic medical records, including sex, birth year, weight, height, and underlying diseases (eg, hypertension and diabetes mellitus). Their signs and symptoms were also obtained from their physician's examination, such as swelling in the wrist and hand, pain severity according to the visual analog scale (VAS), hand numbness/tingling, restricted hand mobility, duration from symptoms onset to admission/diagnosis, and other symptoms of systemic TB infection (eg, fever, weight loss, nocturnal sweating).

Laboratory results were obtained from their medical records, including complete blood count, liver enzymes, urea, creatinine, erythrocyte sedimentation rate (ESR), C-reactive protein (CRP), chest X-ray, hand X-ray, hand ultrasound, interferon-gamma release assay (IGRA), and histopathological features.

Treatment was based on the Vietnam's guidelines with a full course of anti-TB therapy. Treatment of new patients comprises a two-month intensive phase with isoniazid, rifampicin, pyrazinamide, and ethambutol, followed by a continuation phase with rifampicin and isoniazid. Once all participants had completed the TB treatment regimen (after 12 months), the investigator conducted telephone interviews to assess their clinical responses to treatment. Patients were asked about their symptom improvement. A TB specialist determined their treatment outcomes, which were categorized as successful (cured/completed), failed/unsuccessful, and died.

### Consistency of research results

Relevant information was collected via a structured questionnaire, and the researcher recorded the data directly. The laboratory tests were performed using the standard test kit at the University Medicine Center HCMC. One orthopedic specialist collected the histopathology specimens, and one pathologist at Pham Ngoc Thach Hospital-Hospital for Tuberculosis and Lung Diseases read and analyzed the results.

### Statistical analysis

Statistical analyses were performed using the software STATA/MP 14.0 for Windows (Stata Corp LLC, College Station, TX, USA). Binary and categorical variables are presented as percentages and numbers. Continuous variables are presented as mean ± standard deviation (SD) or median and interquartile range. The Kaplan-Meier survival curve was used to assess treatment responses after the follow-up period.

## Results

We enrolled 25 patients with TB tenosynovitis who met the study criteria. Table 1 lists the primary baseline characteristics of the study sample. The mean age was 58.08 ± 16.38 years, ranging from 28 to 83 years. The numbers of men and women were similar, with a 1:1 ratio.

The clinical characteristics of TB tenosynovitis are listed in Table 2. Most patients (75%) were affected in their dominant hand. All parts of the wrist and hand could be affected; the most common location was the wrist and fingers (25%). The flexor tendon is more commonly affected than the extensor tendon (72% vs 28%). The most common symptom was swelling of the wrist and hand, which was present in all patients. While three-quarters of patients had pain symptoms, their pain level on the VAS scale was usually mild to moderate, with an average pain level of 3. In addition to hand swelling and pain, hand numbness occurred in 24% of the TB tenosynovitis patients. All patients had no systemic manifestations of TB infection. The average time from symptom onset to examination was about six months, ranging from one to 18 months.

The subclinical characteristics of the study sample are summarized in Table 3. Complete blood cell analysis showed no abnormalities in blood cell lines such as white blood cells, red blood cells, and platelets. Liver enzymes aspartate aminotransferase (AST), alanine aminotransferase (ALT), and creatinine levels were mostly within normal limits. There were slight increases in inflammatory markers such as ESR (52.4 ± 37.83 mm/h) and CRP concentration (20.63 ± 16.60 mg/L). The IGRA was positive for 40% of patients.

On imaging, 88% of patients had a normal chest X-ray. The observed abnormalities included opacity and fibrous lesions in 8% and 12% of patients, respectively. No abnormalities were noted in the hand X-rays of nearly 90% of patients. All patients had abnormal hand ultrasounds. Soft tissue edema and tendon sheath thickening were the two most common signs, which were present in 88% and 80% of patients, respectively. No patients in our study sample had human immunodeficiency virus (HIV) infections or were rheumatoid factor (RF) positive.

**Figure 1:**
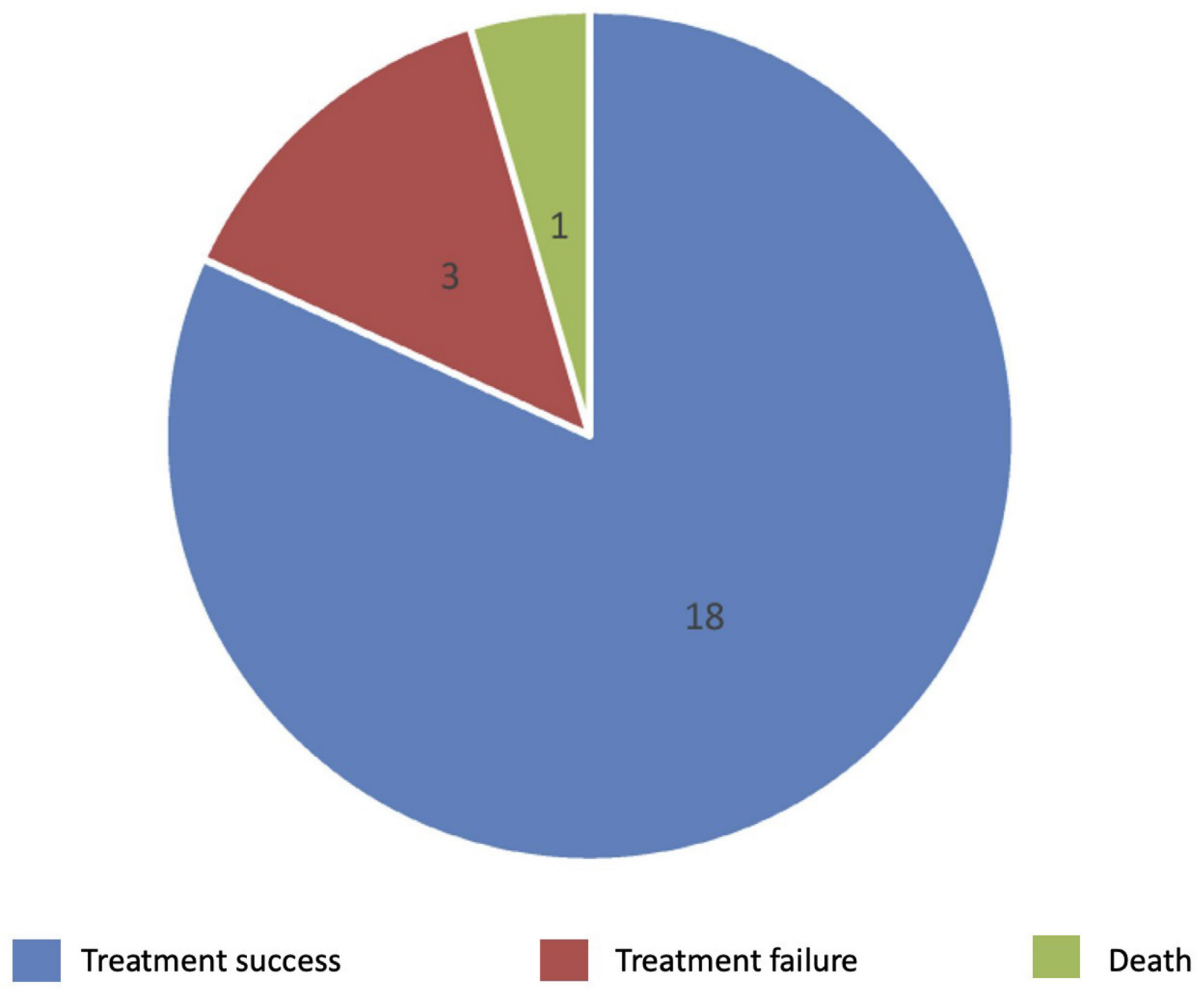
Outcomes of the TB tenosynovitis patients after the treatment period.

Figure 1 provides an overview of the participants' treatment outcomes in the study sample. After the treatment follow-up period, three patients had missing follow-ups. A tuberculous expert determined that 18/22 patients had treatment success. Specifically, 13/18 patients were determined to be in complete recovery, while 5/18 had reduced symptoms but still had mild swelling or slight pain after the treatment course. In addition, 3/22 patients did not respond to the initial treatment regimen; their symptoms continued progressing, and they needed treatment for > 12 months. Moreover, one patient died from TB dissemination after discontinuing treatment after one month because he was intolerant to the drug.

**Figure 2:**
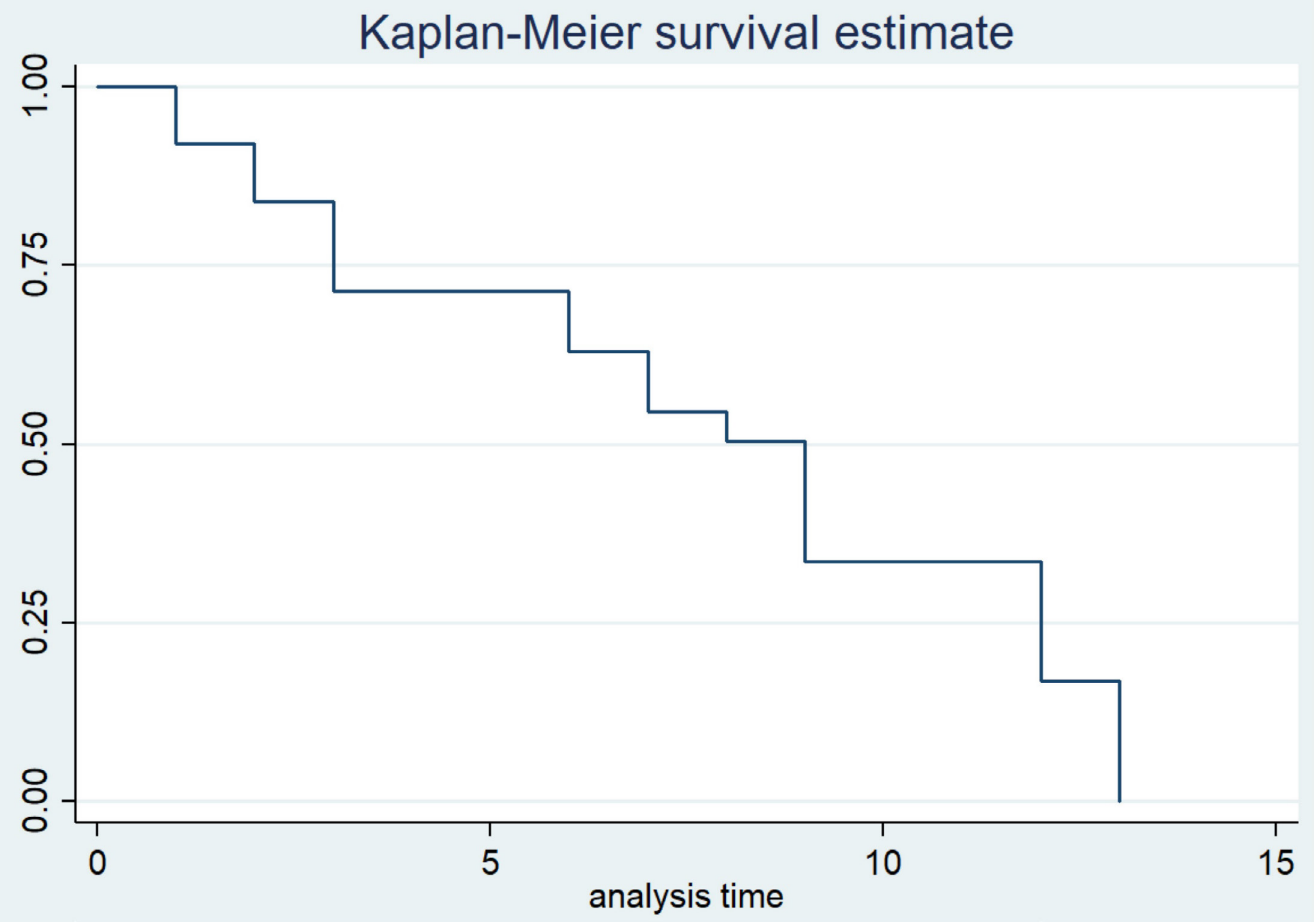
A Kaplan-Meier survival curve of the treatment response in TB tenosynovitis patients.

Figure 2 illustrates the treatment response in TB tenosynovitis patients through the follow-up time. The duration of TB treatment fluctuated from 9 to 12 months. The average time for most patients to see symptom improvement was three months (3.35 ± 1.78). No recurrences were noted in the recovering patients during the follow-up time.

## Discussion

*Mycobacterium tuberculosis* infection is primarily seen in patients with immune deficiency, potentially leading to disseminated infections and a high mortality rate. Several reports have shown that the precipitating factors for TB infection include age >60 years, trauma, joint overuse, malnutrition, alcoholism, low socioeconomic status, TB exposure history, immunosuppression, and long-term corticosteroid treatment.^7^-^10^ There are two hypotheses for TB tenosynovitis pathogenesis: direct infection and hematogenous infection from a primary focus.^11^ Kanavel et al.^12^ stated that tendon sheath TB comprised three stages. The first stage involves serous exudation due to sheath thickening. The second stage is the proliferative phase of granulomatous tissues forming rice bodies. The third stage is necrosis.

Patients with TB tenosynovitis generally have an insidious progression and prolonged disease duration. Our study found that the average duration from symptom onset to examination was 6.08 ± 4.67 months but sometimes up to 18 months. Bayram et al.^13^ also noted that the time to diagnose TB tenosynovitis is often prolonged, ranging from 12 to 18 months. Due to atypical clinical manifestations, many cases of tendon TB in the wrist region have delayed diagnoses and are misdiagnosed as other diseases such as rheumatoid arthritis, gouty arthritis, De Quervain tendonitis, and carpal tunnel syndrome.^6^, ^8^, ^11^ In addition, open biopsy and histopathological specimen culturing are often required for diagnosis. However, this will take time and may contribute to delays in patient diagnosis and treatment. This delay was approximately one month in our study.

Our study found that any part of the wrist and hand can be affected by this disease, with relatively equal frequencies. Besides, compared to extensor tendons, flexor tenosynovitis is much more commonly affected. Sang-Min Lee et al.^14^ also reported that, for unknown reasons, the flexor side is more commonly involved than the extensor side, and the ulnar border is more commonly involved than the radial border.

**Figure 3:**
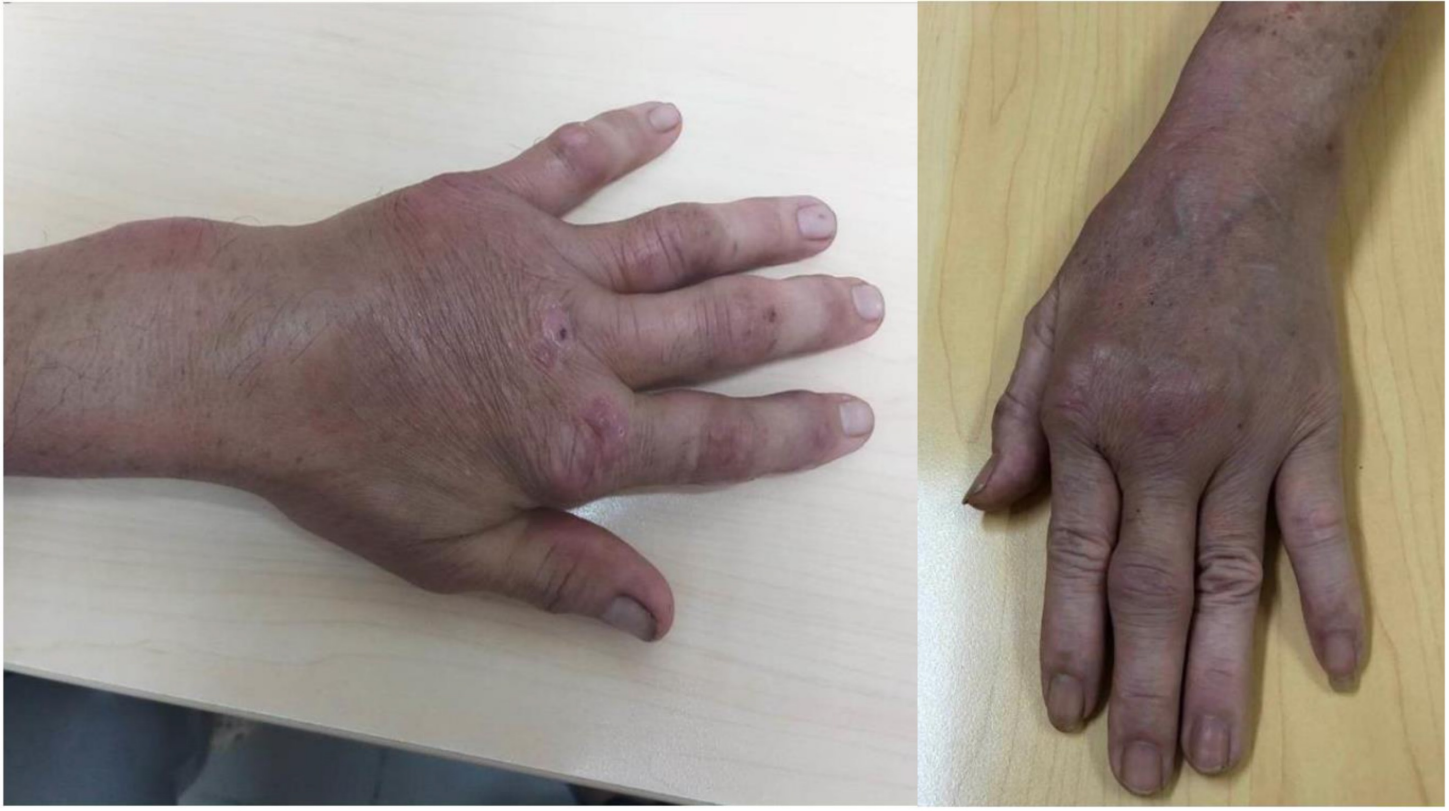
Swelling left hand and arthritis-like pattern in 2 different patients with TB tenosynovitis.

The most common symptom is swelling of the affected area (Figure 3), the main symptom significantly affecting activities. In addition, pain can also be encountered in some patients. It is worth noting that the pain level fluctuated only slightly, with an average VAS of 3.00 ± 2.88. In addition, numbness due to nerve compression may also occur in some patients. These findings are consistent with the literature, where patients with TB tenosynovitis often have three classical clinical presentations: first, swelling in the wrist or hand with mild pain and limited range of motion; second, a sausage-like shape on the influenced fingers; third, numbness and tingling in carpal tunnel syndrome caused by nerve compression in the hand area. However, these symptoms are often nonspecific and can be seen in other pathologies. Because of the gradual onset, most patients present with progressive disease.

**Figure 4:**
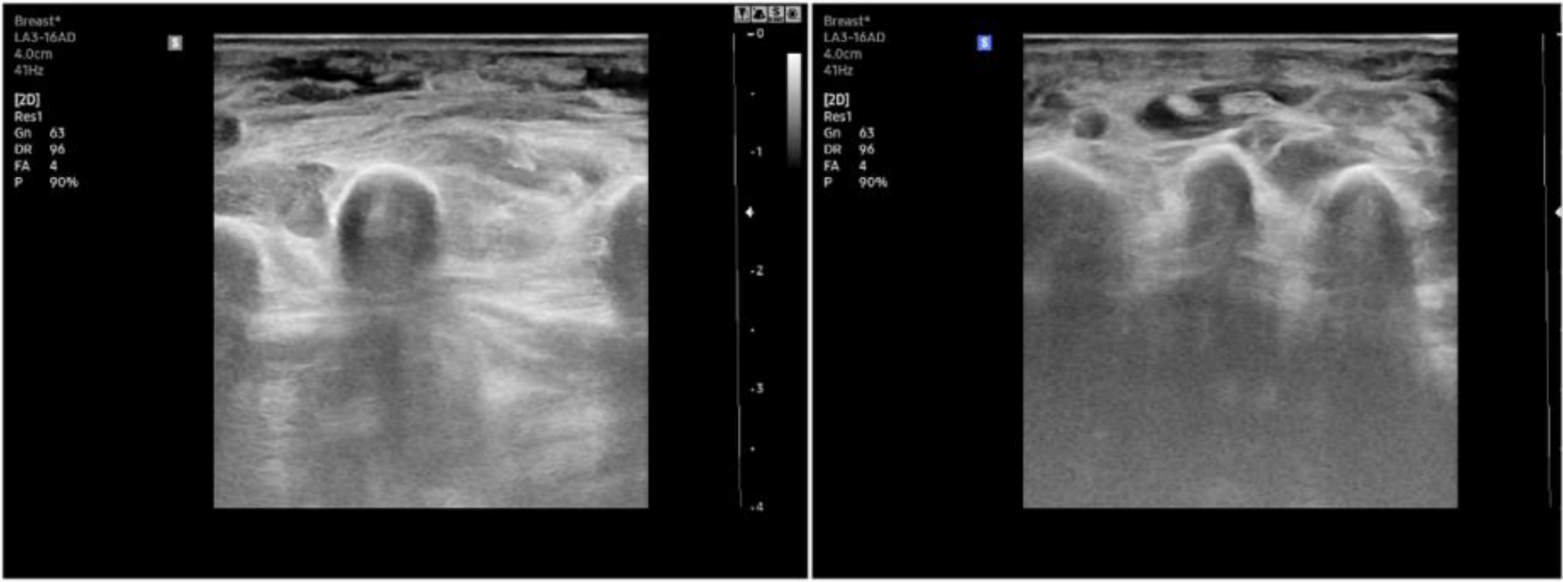
Swelling of soft tissue and peritendinous effusion in hand ultrasounds.

Laboratory studies such as complete blood count and liver and renal functions may be normal or mildly elevated in some patients. However, these parameters are not specific to TB, and inflammatory markers such as ESR and CRP were slightly elevated in most patients due to the body's inflammatory response to the TB infection.^15^ Studies have also noted that hand X-rays are not often supportive of the diagnosis. Abnormal findings, such as bone resorption, were only noted at the late stage. In our study, 88% of patients had no visible abnormalities in their hand X-rays, and some lesions were recorded at a late stage, such as bone resorption and joint space stenosis. Hand ultrasound is a useful and non-invasive tool that can be used to confirm the TB tenosynovitis diagnosis and discover its extension.^8^ All participants in our study had abnormal ultrasounds, with signs such as tendon sheath thickening, tendon sheath fluid accumulation, and soft tissue edema of the hand (Figure 4). Chest X-rays in musculoskeletal TB are primarily normal. Pulmonary TB is present in 50% of TB tenosynovitis patients. However, only a few patients in our study had a lesion on lung imaging. The IGRA is a method to detect latent TB infection.^16^ However, this is not a useful tool for confirming the diagnosis because only 40% of participants in our study had a positive IGRA.

**Figure 5:**
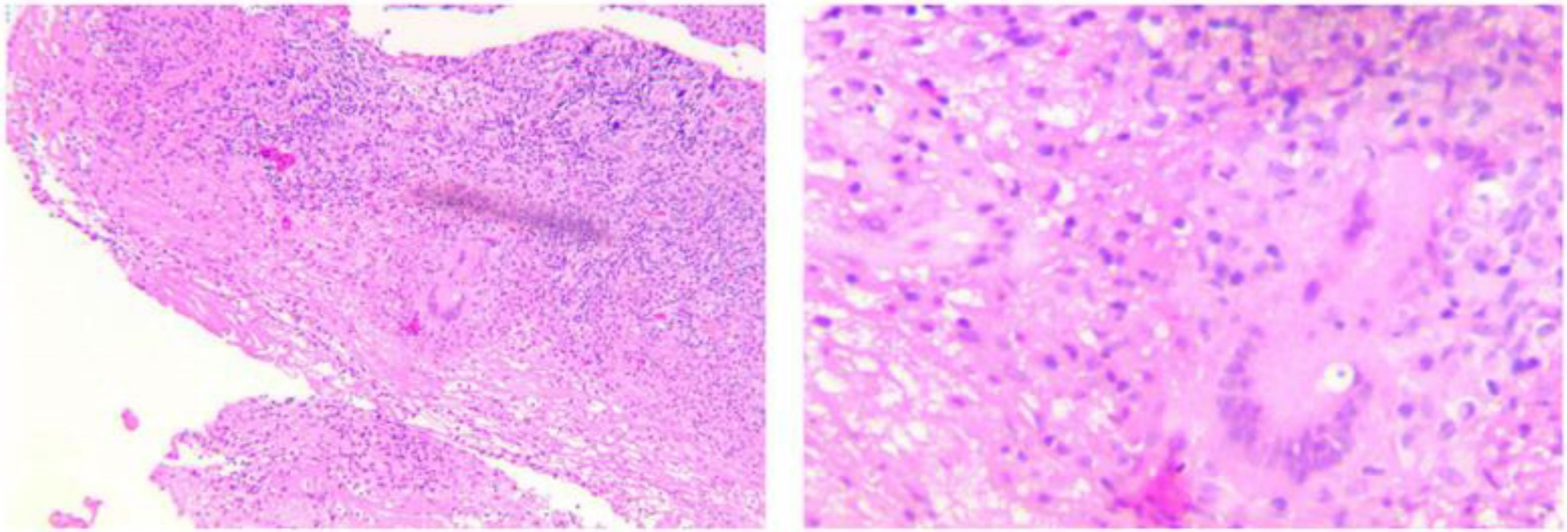
A TB cyst with caseous necrosis surrounded by Langerhans giant cells and lymphocytes.

Microbiologic findings cannot confirm the diagnosis because acid-fast bacillus is only positive in 10% of synovial fluid, 20% of synovial tissue, and 30% of the regional lymph node specimens.^8^ Therefore, negative microbiologic findings cannot help to exclude this disease. Our study used histopathology to confirm the diagnosis through caseous granuloma inflammation and Langerhans giant cells in biopsy specimens (Figure 5).

**Figure 6:**
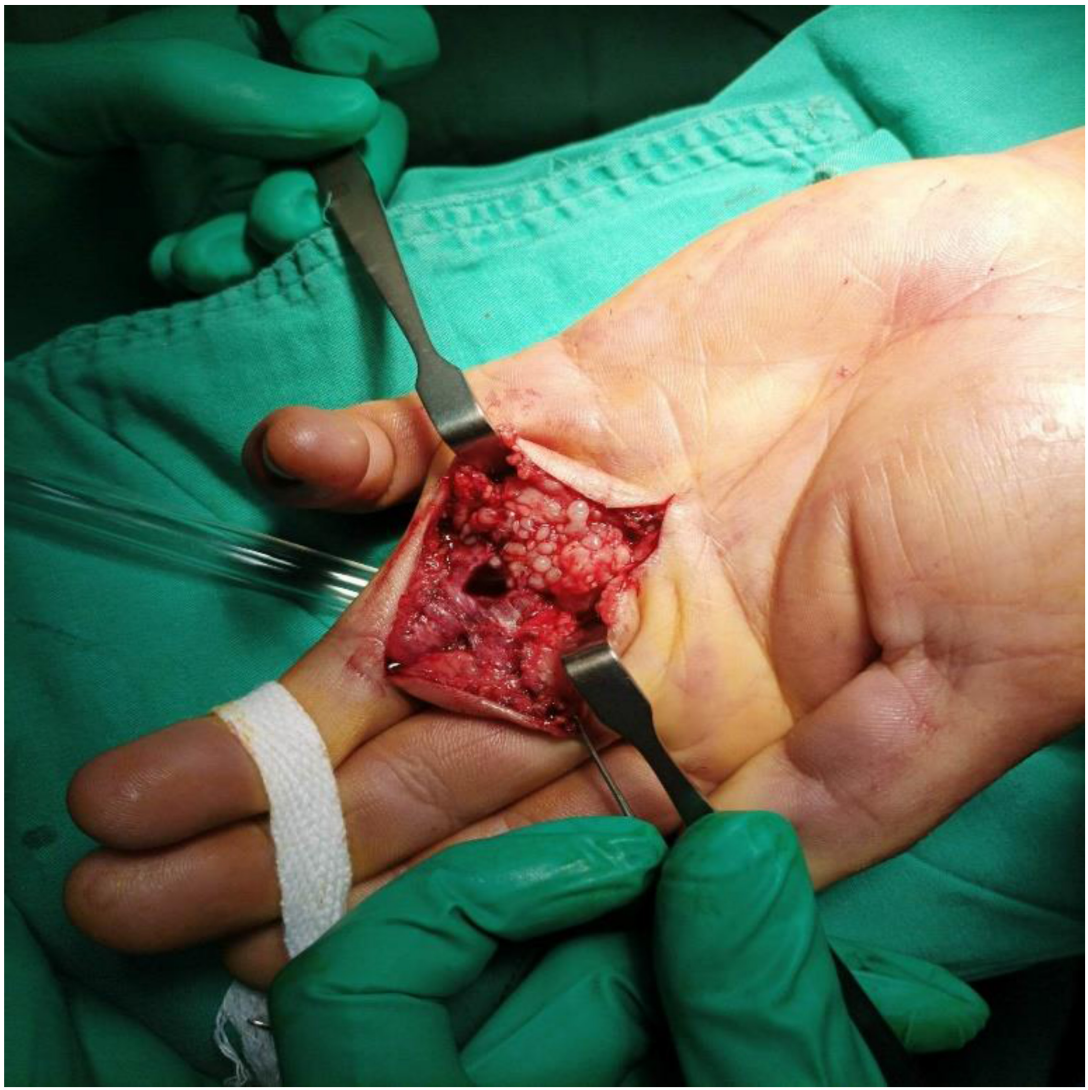
Multiple rice bodies around the flexor tendons in the palm.

It is imperative to initiate treatment with anti-TB drugs as soon as possible after a definite diagnosis because adequate treatment can prevent complications and recurrence. TB tenosynovitis should be treated with a four anti-TB drugs regimen comprising isoniazid, rifampicin, pyrazinamide, and ethambutol for two months, followed by maintenance with isoniazid and rifampicin for at least four months. Some studies have suggested a longer treatment period of up to 18 months.^17^, ^18^ In our study, the treatment course lasted 9-12 months, with one case receiving prolonged treatment for up to 18 months. TB tenosynovitis generally responded to medical treatment in our study. The average time for most patients to see improvement in symptoms was three months (3.35 ± 1.78). One major obstacle that makes these patients abandon treatment is the drugs' adverse effects. Surgery is considered only for biopsy, drainage of extended necrotic lesions, or in patients with nerve compression.^8^, ^19^ The biopsy specimens of some patients in our study contained rice bodies (watermelon seeds) around the tendon (Figure 6) believed to reflect inflammation progression in the tendon sheath.^13^ Woon et al.^20^ reported that operative findings of rice bodies, millet seeds, or melon seeds highly suggest TB tenosynovitis. Regnard et al.^21^ reported that local recurrence was possible, and about 50% of cases recurred within one year of treatment. However, no recurrences were noted in our study.

Our study had several limitations. First, its sample size was small. Second, its limited follow-up time may not fully assess the recurrence rate of the disease. Further research should be undertaken to investigate the complete picture of this in the future. However, to our knowledge, this is the first study addressing the characteristics and treatment outcome of TB tenosynovitis in Vietnam, providing some guidance for evaluating and managing this rare disease.

## Conclusions

TB tenosynovitis is an uncommon presentation that can easily be confused with other diagnoses. Disease progression is often insidious, leading to delays in diagnosis and treatment. Its most common symptoms are swelling of the hand and mild pain. A histological examination is required to confirm the diagnosis. In addition, ultrasound is a useful tool in diagnosing and evaluating tendon sheath injuries. Most cases respond and have a good outcome after 9-12 months of anti-tuberculous treatment. In Vietnam and other endemic TB areas, TB tenosynovitis should be included in the differential diagnosis when a patient presents with chronic wrist or hand swelling.

## Figures and Tables

**Table 1:** Baseline characteristics of the study sample (*n* = 25).

Characteristic	Value
Age, mean ± SD	58.08 ± 16.38
Sex, *n* (%)	
Male	11 (44)
Female	14 (56)
Weight (kg), mean ± SD	55.64 ± 7.63
Height (cm), mean ± SD	160.80 ± 6.92
Body mass index (kg/m^2^), mean ± SD	21.49 ± 2.44
Hypertension, *n* (%)	6 (24)
Type 2 diabetes, *n* (%)	6 (24)
Cushing syndrome, *n* (%)	3 (12)
Taking an immunosuppressive drug, *n* (%)	1 (4)
Pulmonary TB, *n* (%)	1 (4)
Familial TB, *n* (%)	2 (8)

**Table 2:** Clinical characteristics of TB tenosynovitis.

Characteristic	Value
Dominant hand, *n* (%)	18 (72.00)
Affected area, *n* (%)	
Palmer surface	
Wrist	2 (8.00)
Palm Fingers	3 (12.00) 2 (8.00)
Wrist and palm	1 (4.00)
Wrist and fingers	4 (16.00)
Wrist, palm, and fingers	4 (16.00)
Palm and fingers	2 (8.00)
Dorsal surface	
Wirst	1 (4.00)
Fingers	1 (4.00)
Wirst and fingers	2 (8.00)
Wirst, dorsal hand and fingers	1 (4.00)
Dorsal hand and fingers	2 (8.00)
Flexor tenosynovitis, *n* (%)	18 (72.00)
Extensor tenosynovitis, *n* (%)	7 (28.00)
Swelling, *n* (%)	25 (100)
Pain, *n* (%)	18 (72.00)
Pain severity, mean ± SD	3 ± 2.88
Numbness/tingling, *n* (%)	6 (24.00)
Limited range of motion, *n* (%)	12 (48.00)
Systemic tuberculous symptoms (eg, fever, weight loss, and night sweats), *n* (%)	0
Duration from symptom onset to admission, mean ± SD (range)	6.08 ± 4.67 (1-18)
Duration from symptom onset to diagnosis, mean ± SD (range)	7.34 ± 4.73 (2-19)

**Table 3:** Laboratory and imaging characteristics in TB tenosynovitis.

Characteristic	Value
White blood cell count (g/L), mean ± SD	11.04 ± 3.48
Neutrophil count (g/L), mean ± SD	7.84 ± 3.18
Lymphocyte count (g/L), mean ± SD	2.31 ± 0.99
Hemoglobin (g/L), mean ± SD	130.00 ± 12.58
Hematocrit (%), mean ± SD	39.90 ± 3.99
Platelet count (g/L), mean ± SD	339.76 ± 95.95
AST (U/L), mean ± SD	25.24 ± 12.95
ALT (U/L), mean ± SD	26.32 ± 13.59
Creatinine (mg/dL), mean ± SD	0.82 ± 0.21
ESR (mm/h), mean ± SD	52.4 ± 37.83
CRP (mg/dL), mean ± SD	20.63 ± 16.60
IGRA positive, *n* (%)	10 (40)
Chest X-ray findings, *n* (%)	
Ground glass opacity	2 (8)
Fibrosis	3 (12)
Normal	22 (88)
Hand X-ray findings, *n* (%)	
Bone erosion	3 (12)
Joint space narrowing	1 (4)
Normal	22 (88)
Hand ultrasound findings, *n* (%)	
Synovial membrane thickening	20 (80)
Peritendinous effusion	16 (64)
Soft tissue swelling	22 (88)
RF positive, *n* (%)	0
HIV infection, *n* (%)	0
